# Non-response bias in estimates of HIV prevalence due to the mobility of absentees in national population-based surveys: a study of nine national surveys

**DOI:** 10.1136/sti.2008.030353

**Published:** 2008-07-22

**Authors:** M Marston, K Harriss, E Slaymaker

**Affiliations:** Centre for Population Studies, London School of Hygiene and Tropical Medicine, London, UK

## Abstract

**Objectives:**

To measure the bias in national estimates of HIV prevalence in population-based surveys caused by mobility and refusal to test.

**Methods:**

Data from nine demographic and health surveys and AIDS indicator surveys were used. Non-responders were divided into three groups: (i) “refusals” who were interviewed but not tested; (ii) “refusals” who were present in the household but not interviewed or tested; and (iii) “absentees” who were absent from the household. Correction for HIV status was made for the non-responders using multiple imputation methods with logistic regression models based on a common set of household-level and individual-level sociodemographic and behavioural factors for those tested and stratified by mobility status.

**Results:**

The non-response groups were corrected to have higher risks of HIV than those who participated in the HIV tests, although these were only detected to be statistically significant in some of the countries. In Lesotho, the corrected prevalence for the absent household members was significantly higher than for those who were present in the household. However, the adjusted prevalences differed by less than a percentage point from the prevalences observed among those who were tested, so the overall effects of non-response on national estimates of HIV prevalence are minimal.

**Conclusions:**

The results indicate that the mobility of absentees does not substantially bias estimates of HIV prevalence from population-based surveys. None the less, if levels of non-response are high or if non-responders differ greatly from those who participate in HIV testing with respect to HIV status, non-response could still bias national estimates of HIV prevalence.

Accurate HIV prevalence estimates are essential for monitoring the epidemic, planning programmes and evaluating interventions. The best data source and method for estimating HIV prevalence are still subject to discussion.

Population-based surveys are a relatively recent source of HIV data. Since the mid-1980s, facility-based sentinel surveillance has been the mainstay for monitoring the HIV epidemic.

HIV testing among pregnant woman was demonstrated to be a good proxy for prevalence in the general population.^1^ The representativeness of ANC data varies by the stage of the HIV epidemic, by age group and region.^2^ ^3^ Adjustment procedures have been developed to control for biases in ANC data.^4^^–^6 Concerns about the limitations of ANC-based surveillance systems and demand for better data led to inclusion of HIV testing in national population-based surveys.^7^ ^8^ These can provide representative estimates of HIV prevalence for the general population as well as for different subgroups—for example, regions, urban and rural areas, by sex and age group. HIV status can be linked to detailed social-demographic and behavioural information. However, population surveys have their own limitations and biases.

- Low-level and concentrated epidemics: household-based surveys underestimate HIV prevalence when infection is concentrated in groups that are inadequately sampled (for example, people living in hostels, prisons, military barracks, refugee camps or brothels).^7^
- Non-response can bias population-based estimates of HIV prevalence if it is systematically associated with HIV. This could occur for two reasons: (i) refusal to participate in HIV testing or (ii) absence from the household at time of survey.^9^ ^10^

Little is known about the association between HIV infection and survey refusal. Testing refusal is presumed to be based on personal risk perception, which has been shown to be weakly or moderately linked to HIV status.^11^ Some respondents may know their HIV status and this may affect participation. Several studies used sociodemographic and behavioural characteristics to predict HIV status among refusers and suggest it is higher than among those who tested.^10^ ^12^

Absence from the household may be another systematic source of bias. People making frequent journeys may not be available when the survey team visits the household.^9^ Increased prevalence of HIV in mobile groups has been frequently reported in sub-Saharan Africa, and plays a key part in the spread of the epidemic (see among others references 13–20).

HIV prevalence estimates can be adjusted for non-response by making assumptions about the ratio of prevalence in respondents and non-respondents. Assuming that non-responders have twice the HIV prevalence of those who participate in surveys, Garcia-Calleja and colleagues calculated that individual non-response could result in an adjusted HIV prevalence 1.03–1.34 times higher than the observed prevalence.^12^

Analysis of Kenyan data concluded that HIV prevalence did not differ in non-responders.^21^ The adjusted prevalence for women in Kenya was slightly lower than the unadjusted prevalence, while the adjusted and unadjusted prevalence rates for men were about the same.^9^ In Ghana and Malawi, similar analyses suggested that adjusted prevalence for non-tested respondents was higher than for tested respondents.^22^ Mishra and colleagues examined the impact of non-response in five demographic and health surveys (DHS) and showed that predicted prevalence among non-responders was, on average, 12% higher than observed prevalence among tested respondents. However, the relatively small numbers of non-responders meant the adjustment made little difference to the estimated national prevalence.^10^

Further improvements in adjustment techniques are needed.^12^ The methods recommended by WHO/UNAIDS and Mishra and colleagues treat absentees like those who accepted testing, with respect to the relation between HIV status and sociodemographic characteristics. This adjustment procedure is unsatisfactory for absentees because evidence from numerous studies suggests that people who are more mobile have higher rates of risky sexual behaviour and are at higher risk of HIV infection. Adjustment methods that ignore the additional risk entailed by mobility may underestimate the prevalence of HIV among absentees.^28^ Here we look for bias in estimates of HIV prevalence in nine DHS using adjustment procedures that are similar to those recommended by WHO and UNAIDS,^9^ except that the HIV status of absentees is modelled on respondents who were interviewed, tested and also mobile.

## METHODS

### Data sources

We used data from eight DHS and one AIDS indicator survey (AIS)^24^ out of the 22 DHS and AIS that have so far included HIV testing. Three surveys were excluded because the HIV test results could not be linked to information in the household and individual questionnaires. Four surveys did not collect data on mobility. Seven surveys were excluded as they did not provide sufficient sample size to carry out the analysis. Of the remaining nine surveys, four did not collect mobility information for women, so only men were analysed.

HIV testing follows the main DHS sample design which is usually two-stage geographically stratified and clustered. At the second stage, a random sample of households is taken, following a full household listing in each cluster. The listing excludes people living in institutions. Selected households complete the household questionnaire, on characteristics of the household’s dwelling and list their usual members and visitors present at the time of the survey. Women aged 15–49 years are eligible for the detailed individual interview. In a subsample of households, men in the target age range (15–49, 15–54 or 15–59) are also interviewed. In this subsample of households, eligible men and women were also requested to provide samples of blood or saliva for HIV testing.^25^ A household member is considered absent if they are not contacted by the survey team after three call-backs. The AIS methods are similar, but the sample is not designed to provide estimates at regional or provincial levels.

### Analysis

The patterns of non-response were complex, because of the repeated visits made to households. Non-responders were categorised as follows:

- “Refusals”, interviewed but not tested: Eligible individuals who completed the individual interview, but refused the HIV test or did not produce a valid test result for other reasons. We termed all those who were interviewed but not tested “refusals”, since respondents who consented to test but then absented themselves may logically be classed as refusals.
- “Refusals”, not interviewed: Eligible individuals present in the household at the time of the survey, who failed to complete an individual interview. For simplicity of interpretations we have also termed these individuals “refusals”. They must be considered separately because HIV status can only be predicted on the basis of the information collected in the household questionnaire. Fieldwork teams usually make three callback visits to the household before designating an individual as not interviewed.^26^ This means a small minority of those not interviewed participated in HIV testing. For these respondents, HIV test data were available without the corresponding interview data.
- “Absentees”, eligible individuals who were listed as usually resident household members, but who slept away the night before the survey and were therefore absent for the interview. After the testing callback visits, a number of those absent for interview eventually participated in HIV testing. The category of “absentee” thus contains a diverse group of mobile people, some of whom returned to the household during the period of fieldwork.

**Figure 1:**
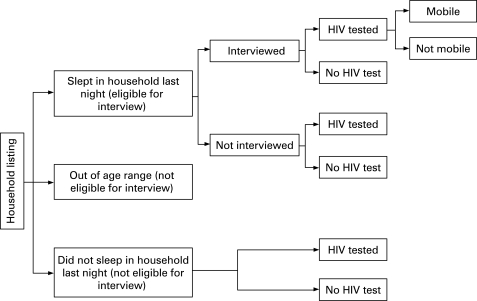
Schematic diagram to show the data available for the different groups of people listed on the household schedule.

Figure 1 shows how the non-responder categories relate to the survey design.

To evaluate the effect of non-response bias on the survey estimates, HIV prevalence was corrected among the three non-response groups using multiple imputation with multiple regression models^27^ ^28^ based on those who were tested, using a common set of household and individual-level predictor variables.

As table 1 illustrates, HIV status among the “refusals” who were interviewed but not tested was corrected using information from respondents who were interviewed and tested. HIV status of the refusals could be corrected using the full range of variables from the survey. HIV status of the “refusals” who were not interviewed or tested was corrected using information from the respondents who were interviewed and tested, and those who were tested despite not being interviewed. HIV status of refusals who were not interviewed was corrected using only household variables, as these respondents lacked the corresponding individual-level data. Absentees were treated as mobile people. Their HIV status was corrected using information from respondents who were interviewed, tested, and reported having undertaken at least one trip in the previous year, to incorporate the excess HIV risk known to be associated with mobility. Apart from mobility, HIV status was corrected using only household variables, as absentees lacked all the other individual-level data.

**Table 1:** Variables used in the correction models for the different groups of household members

Group number	1	2	3	4	5	6	7
Outcome of interview and HIV testing	Interviewed tested mobile	Interviewed tested non-mobile	Interviewed not tested (refusal)	Not interviewed tested	Not interviewed not tested (refusal)	Absent tested	Absent not tested
Corrected using groups:			1+2		1,2 and 4		1
Household-level variables in correction models			Yes		Yes		Yes
Individual-level variables in correction models			Yes		No		Only the “mobility” variable

Mobility was identified from two items in the individual questionnaire. Mobile people were defined as those who had one or more trips outside their home community in the previous year. We expanded the mobility definition to include respondents who logically should have been classed as mobile, even though some did not actually answer positively to the question about trips. This included respondents who had made any trip in the last year; long-term migrants; and visitors (that is, individuals present in the household during the survey but not usually resident).

The household predictors for prevalence in all non-response groups were: age, education, wealth index (based on household assets),^29^ urban/rural residence and geographical region. These were available for every country.

To correct HIV status for the “refusals” who were interviewed but not tested, we used a common set of individual-level sociodemographic and behavioural factors, as recommended by WHO and UNAIDS.^9^ ^10^ In addition to migration, individual-level variables included marital status, childbirth in the last 5 years (women only), work status, media exposure (frequency of using newspapers, television and radio); religion, circumcision, sexually transmitted disease (STD) in the last year, alcohol use, cigarette smoking, age at sexual debut, number of sexual partners in the last year, condom use at last sex in the last year, paid for sex (men only), higher-risk sex in the last year (that is, sex with a non-marital, non-cohabiting partner). Variables included in the final models vary by country because data were not available for every country or because sample size was insufficient.

Adjusted HIV prevalence was calculated by summing observed prevalence among those who were tested, and the corrected prevalence in the groups of non-responders. All analyses were carried out separately for men and women. Unless otherwise specified, statistical significance relates to a 0.05 probability level.

Data analysis was carried out using the statistical package Stata 10.0 SE. Clustering in the survey design was taken into account in all the analyses. Household weights were used for all the analyses except when calculating HIV prevalence of those who tested, where we used the HIV weights to ensure that the results corresponded with the prevalence reported by DHS.

## RESULTS

### Response rates

Table 2 shows response rates by HIV and mobility status and reasons for non-response. Individual response rates were higher among women than men. The proportion of male respondents who were both interviewed and tested ranged from 44% in Lesotho to 92% in Rwanda. Among women, response rates varied from 63% in Lesotho to 95% in Rwanda. Of those interviewed and tested, the proportion who had made at least one trip in the past year ranged from 18% to 56% for women and 25% to 57% for men. The largest category of non-responders was the “refusals” who were interviewed but not tested. The lowest refusal rates were recorded in Rwanda, and the highest in Malawi (because of technical problems fielding the survey in Lilongwe, as reported in the Malawi demographic and health survey^22^). The percentage of “refusals” who were not interviewed or tested ranged from a minimum of 1.8% of women and 2.7% of men in Rwanda, to 9.4% of women in the Cote d’Ivoire and 17.3% of men in Zimbabwe. The percentage of respondents who were absentees ranged from 1.8% of women and 3.6% of men in Rwanda to a maximum of 21% of women and 35% of men in Lesotho. The number of visitors who were tested ranged from 40 in Ghana to 273 in Côte d’Ivoire.

**Table 2:** Numbers and percentages (unweighted) of eligible respondents by interview and HIV test outcome and mobility (defined as making at least one trip away from home in the last year)

Country	Total	1	2	3	4	5	6	7
		Interviewed and tested mobile	Interviewed tested non-mobile	Interviewed not tested (refusals)	Not interviewed tested	Not interviewed not tested (refusals)	Absentees Tested	Absentees not tested
		No (%)	No (%)	No (%)	No (%)	No (%)	No (%)	No (%)
**Women**								
Cote d’Ivoire	6236	1567 (25.1)	2961 (47.5)	648 (10.4)	0 (0.0)	589 (9.4)	56 (0.9)	415 (6.7)
Ethiopia	7439	1086 (14.6)	4855 (65.3)	870 (11.7)	11 (0.1)	319 (4.3)	111 (1.5)	187 (2.5)
Haiti	5430	1334 (24.6)	3889 (71.6)	76 (1.4)	21 (0.4)	47 (0.9)	53 (1.0)	10 (0.2)
Rwanda	5942	1293 (21.8)	4370 (73.5)	66 (1.1)	14 (0.2)	94 (1.6)	60 (1.0)	45 (0.8)
Zimbabwe	10195	4195 (41.1)	3289 (32.3)	1409 (13.8)	0 (0.0)	963 (9.4)	0 (0.0)	339 (3.3)
**Men**								
Cote d’Ivoire	5582	1794 (32.1)	2098 (37.6)	610 (10.9)	0 (0.0)	645 (11.6)	72 (1.3)	363 (6.5)
Ethiopia	7337	1618 (22.1)	3483 (47.5)	923 (12.6)	11 (0.1)	734 (10.0)	200 (2.7)	368 (5.0)
Ghana	5743	2424 (42.2)	1841 (32.1)	750 (13.1)	6 (0.1)	324 (5.6)	100 (1.7)	298 (5.2)
Haiti	5209	1605 (30.8)	3209 (61.6)	142 (2.7)	22 (0.4)	114 (2.2)	94 (1.8)	23 (0.4)
Kenya	4377	1422 (32.5)	1495 (34.2)	661 (15.1)	24 (0.5)	581 (13.3)	84 (1.9)	110 (2.5)
Lesotho	5110	925 (18.1)	1288 (25.2)	560 (11.0)	12 (0.2)	496 (9.7)	33 (0.6)	1796 (35.1)
Malawi	4017	895 (22.3)	1509 (37.6)	857 (21.3)	0 (0.0)	536 (13.3)	54 (1.3)	166 (4.1)
Rwanda	5135	1196 (23.3)	3523 (68.6)	92 (1.8)	14 (0.3)	125 (2.4)	113 (2.2)	72 (1.4)
Zimbabwe	9180	2954 (32.2)	2599 (28.3)	1617 (17.6)	0 (0.0)	1586 (17.3)	0 (0.0)	424 (4.6)

### Mobility and HIV risk

After controlling for household and individual-level risk factors, there was a non-significant but positive association between mobility and HIV risk for men and women in almost all countries. For men in Lesotho the association was statistically significant (OR 1.83, CI 1.01 to 3.32). Risks associated with mobility varied according to age. In all countries except for Zimbabwe males, there was a trend for younger mobile people to be disproportionately more at risk of HIV than older mobile people, but this was not statistically significant.

### Correction of HIV in the non-response groups and adjustment of national prevalence

Table 3 presents corrected prevalence for non-response groups showing how they differed from observed HIV prevalence among tested respondents, and the impact that non-response bias had on national adjusted prevalence.

**Table 3:** Prevalences, from observed (tested), corrected and adjusted methods with 95% confidence intervals

Prevalence	Country, sex, age													
	Cote d’Ivoire		Ethiopia		Ghana	Haiti		Kenya	Lesotho	Malawi	Rwanda		Zimbabwe	
	Men (15–49)	Women (15–49)	Men (15–59)	Women (15–49)	Men (15–59)	Men (15–59)	Women (15–49)	Men (15–54)	Men (15–59)	Men (15–54)	Men (15–59)	Women (15–49)	Men (15–54)	Women (15–49)
1 All HIV tested respondents	2.86 (2.06 to 3.66)	6.41 (5.41 to 7.41)	0.98 (0.66 to 1.31)	1.86 (1.38 to 2.33)	1.66 (1.23 to 2.10)	2.11 (1.54 to 2.68)	2.32 (1.86 to 2.78)	4.71 (3.76 to 5.65)	18.99 (16.94 to 21.03)	10.18 (8.57 to 11.80)	2.24 (1.79 to 2.69)	3.65 (3.14 to 4.16)	14.75 (13.46 to 16.05)	21.12 (19.66 to 22.58)
2 All HIV tested and present	2.95 (2.13 to 3.76)	5.90 (4.92 to 6.87)	0.88 (0.57 to 1.19)	1.65 (1.20 to 2.09)	1.60 (1.19 to 2.01)	2.14 (1.56 to 2.71)	2.31 (1.84 to 2.77)	4.69 (3.69 to 5.69)	18.58 (16.63 to 20.53)	9.69 (8.19 to 11.19)	2.23 (1.77 to 2.68)	3.61 (3.10 to 4.12)	14.47 (13.23 to 15.72)	21.37 (20.06 to 22.49)
3 Those present but not tested, refusals (corrected)	3.74 (1.13 to 6.35)	8.62 (4.78 to 12.46)	1.78 (0.16 to 3.39)	4.01 (0.48 to 7.53)	1.95 (1.32 to 2.16)	2.53 (0.00 to 6.55)	2.98 (0.00 to 9.83)	5.29 (2.98 to 7.59)	21.04 (16.15 to 25.93)	11.43 (7.41 to 15.45)	2.63 (0.00 to 6.77)	4.86 (0.00 to 10.73)	16.67 (14.01 to 19.33)	21.06 (17.83 to 24.30)
4 Ratio present and not tested/present and tested	1.27	1.46	2.02	2.43	1.22	1.19	1.29	1.13	1.13	1.18	1.18	1.35	1.15	0.99
5 Adjusted prevalence for all present	3.11 (2.20 to 4.03)	6.38 (5.30 to 7.46)	1.01 (0.67 to 1.34)	1.83 (1.38 to 2.28)	1.69 (1.21 to 2.17)	2.16 (2.06 to 2.34)	2.34 (2.28 to 2.44)	4.65 (3.75 to 5.54)	19.08 (17.19 to 20.97)	10.03 (8.52 to 11.55)	2.25 (2.19 to 2.38)	3.65 (1.79 to 2.70)	15.10 (13.98 to 16.22)	21.27 (20.06 to 22.49)
6 All not present,absentees (corrected)	3.83 (0.38 to 7.72)	7.49 (2.86 to 12.12)	2.39 (0.26 to 4.51)	2.36 (0.00 to 5.441)	1.62 (0.00 to 3.72)	4.55 (0.00 to 10.23)	1.80 (0.00 to 6.51)	5.57 (0.79 to 10.35)	22.82 (19.01 to 26.63)	9.87 (3.37 to 16.37)	2.62 (0.00 to 5.27)	6.21 (1.22 to 11.21)	15.46 (9.55 to 21.36)	20.86 (13.85 to 27.87)
7 Ratio not present/present and tested	1.23	1.17	2.36	1.29	0.96	2.11	0.77	1.20	2.12*	1.02	1.18	1.72	1.07	0.98
8 All in eligible households	3.04 (2.32 to 4.68)	6.57 (5.00 to 10.48)	1.09 (0.75 to 1.42)	1.89 (1.45 to 2.34)	1.70 (1.22 to 2.17)	2.19 (1.63 to 2.75)	2.34 (1.87 to 2.80)	4.76 (3.86 to 10.35)	20.53 (18.74 to 22.31)	10.27 (8.83 to 11.71)	2.24 (1.79 to 2.69)	3.66 (3.15 to 4.17)	15.12 (14.01 to 16.23)	21.20 (20.04 to 22.35)
9 Ratio: all in eligible households/all HIV tested respondents	1.06	1.02	1.10	1.01	1.02	1.04	1.01	1.01	1.08	1.01	1.00	1.00	1.02	1.01

*p<0.05.

In table 3, DHS estimates of HIV prevalence, given in the first row, include all respondents who were tested, including those who were absent at the time of the interview but were later tested during callback visits. The second row shows observed HIV prevalence only among those who were present in the household at the time of the interview. Exclusion of tested absentees (and visitors as they are mobile people) brings down observed HIV prevalence in all countries, reflecting the fact that tested absentees have a higher risk of HIV than those who were present for interview and tested.

The next rows give corrected prevalence in the non-response groups and adjusted national prevalence. From row 3 we can see that corrected prevalence for “refusals” (both those interviewed but not tested, and those neither interviewed nor tested) is higher than observed prevalence for men and women in most countries. The ratios in row 4 show that corrected HIV status of the “refusals” was relatively high compared with those who were present and tested. These ratios ranged from 0.99 for women in Zimbabwe to 2.43 for Ethiopian women. Row 5 shows that adjusting for corrected higher HIV prevalence in refusals raises national HIV prevalence, but only slightly. At most, for women from Cote d’Ivoire, it made a difference of 0.8% to overall national HIV prevalence (from 5.9% to 6.4%), and confidence intervals around the two estimates overlapped completely.

Corrected prevalence for absentees is given in row 6. In all countries apart from Haiti (females) and Ghana (males), absentees are corrected to have a higher prevalence of HIV than those who were present and tested. Ratios in row 7 show that higher HIV status of absentees compared to those who were present was only statistically significant for Lesotho (2.12 times higher, p<0.01), where there were large number of absentees. Elsewhere there may be insufficient power to detect associations. Comparing rows 4 and 7 shows that relative prevalence of HIV is equally high for both non-response groups – “refusals” and absentees—compared with tested groups. Finally, row 8 shows that adjusting national prevalence by accounting for the corrected HIV status of non-responders made little difference to the estimates. In all countries the adjusted national prevalence is slightly higher than the observed prevalence, but adjustment only raised national prevalence by a maximum of 1.5% (in Lesotho). Row 9 presents ratios of adjusted to observed prevalences. All prevalences remained the same or were adjusted upwards, adjusted prevalence was at most 1.10 times higher than observed prevalence, and this difference was not statistically significant for any country.

## DISCUSSION

This study examined mobility as a source of non-response bias in estimates of HIV prevalence from population-based surveys. We found that data requirements for performing this type of adjustment are not widely met in DHS and AIS surveys and we could use only nine of the 22 available surveys. DHS are not primarily designed to estimate HIV prevalence, and the sample size may be inadequate where HIV prevalence is low, which limits the scope for adjustment for non-response. In sub-Saharan African countries with high prevalence and generalised HIV epidemics, there may be sufficient numbers of HIV-positive respondents for a meaningful analysis, although even in high-prevalence countries the statistical power is low after stratifying by mobility. Larger sample sizes are required to model variation in risk more precisely.

The scope for adjustments was also limited by paucity of information available in non-response groups. There were large numbers of non-responders whose HIV status could only be corrected on the basis of information from household questionnaires. Roughly a third of the non-responders was eligible individuals present in the household during the survey but for a variety of reasons did not complete an individual interview. Additional information would need to be collected in the household questionnaire on characteristics of those who are not interviewed to facilitate more complete adjustment for non-response.

We have confirmed that mobility is an important correlate of HIV infection, although significant associations were only detected in some countries. Associations between mobility and HIV risk are complex, and vary from country to country. In keeping with previous research, multiple regression analyses showed that mobile people were more at risk of HIV. Adjusting for the additional HIV risk of mobile people who are absent from the survey requires large samples of migrants to allow the complex associations between mobility and HIV risk to be mapped out, detected as significant and used for adjustments.

Our investigations showed that some mobile people are captured by the DHS. Absent household members who are present in another household are equally likely to be sampled as those who remain at home. In the DHS, visitors are interviewed and subject to HIV testing even though they are not at their usual place of residence. The number of male visitors in the survey ranges from 17% of the number of absentees in Ghana to 80% in Cote d’Ivoire. For women, numbers of visitors were closer to number of absentees, ranging from 78% in Zimbabwe to 98% in Rwanda. Small numbers prevented us from comparing HIV prevalence among mobile people interviewed as visitors in another household with those who stayed at home. Callback visits ensure that some eligible individuals who are initially absent may eventually be contacted by the survey team for HIV testing, even though this cannot be linked to full individual-level information because they did not complete an interview. Mobility only becomes a major source of non-response bias when substantial numbers of people are not present in any household during the survey.

In common with other research,^10^ ^30^ the corrected prevalence in non-response groups in all countries was found to be higher than observed prevalence in those who were tested. However, given the size of the datasets and lack of statistical power, differences between corrected prevalence in refusals and prevalence in usual residents who were tested were not significant. Similarly, corrected prevalence among absentees was only significantly higher than in tested usual residents in Lesotho. Adjustments to national HIV prevalence estimates showed that accounting for corrected rates among non-responders made little difference to overall estimates. Even in Lesotho, where 55% of eligible men were not tested for HIV, the adjusted prevalence of 20.5% only differed by one and a half percentage points from the observed national prevalence of 19.0%.

This study presents no evidence that mobility of absent household members is an important source of non-response bias in population-based surveys. In all countries except Lesotho, refusal was a more common source of non-response than absence. Non-response because of absenteeism will only have a significant effect on estimates of national prevalence if levels of absenteeism or the relative risk among absentees is substantial. This finding supports other work concluding that population-based surveys do not drastically underestimate HIV prevalence. However, it is important to question the extent to which we are able to predict HIV risk among the absentees based on the HIV risk of mobile people identified by the survey questionnaire items. In Lesotho, it is likely that HIV risk of the mobile men captured by the survey is different from the 35% of eligible men who were absent from the households; absentees were mostly labour migrants elsewhere in Lesotho or in other countries. If absentees are atypical of mobile people surveyed, the assumptions used in the corrections are undermined. This is also true of the assumptions used to correct the status of those people who were interviewed but not tested.

Despite the fact that adjustments made little difference to national estimates, it remains important to examine characteristics of non-responders, especially if rates of non-response are high. The importance of non-response as a source of bias may differ according to survey implementation. In Malawi, for example, mis-fielding of the survey in Lilongwe led to unusually high levels of non-response and the adjusted HIV results for Lilongwe raised the prevalence in the city from 3.7% to 10.8%.^22^

In conclusion, this study tends to support other work showing that national estimates of HIV prevalence provided by population-based surveys are not substantially compromised by mobility and non-response biases.
